# Effects of dog ownership on the gut microbiota of elderly owners

**DOI:** 10.1371/journal.pone.0278105

**Published:** 2022-12-07

**Authors:** Chaona Jiang, Zeying Cui, Pingming Fan, Guankui Du

**Affiliations:** 1 Morphology laboratory, Hainan Medical College, Haikou, China; 2 Department of Breast-Throcic Tumor Surgery, The First Affiliated Hospital of Hainan Medical University, Haikou, China; 3 Key Laboratory of Molecular Biology, Hainan Medical University, Haikou, China; 4 Department of Biochemistry and Molecular Biology, Hainan Medical University, Haikou, China; 5 Biotechnology and Biochemistry Laboratory, Hainan Medical University, Haikou, People’s Republic of China; Hunan Agricultural University, CHINA

## Abstract

Dog owners are usually in close contact with dogs. Whether dogs can affect the gut microbiota of elderly dog owners is worth studying. Data from 54 elderly (over 65 years of age) dog owners were screened from the American Gut Project. Owning a dog did not affect the α-diversity of the gut microbiota of the dog owner. Dog ownership significantly modulated the composition of the gut microbiota of the dog owner. The abundance of *Actinobacteria* was significantly increased. The abundances of *Bifidobacteriaceae* and *Ruminococcaceae* were significantly increased, while the abundance of *Moracellaceae* was significantly suppressed. In general, dog ownership can regulate the composition of gut microbiota and has a more significant effect on elderly males.

## Introduction

Dogs are one of the most important companions of human beings [1]. Keeping a dog can promote communication between people. The owner can release pressure and relieve a depressed mood by raising a dog [2]. The social and emotional health status of children in families with a dog is better than that of children in families without a dog [3]. Studies have shown that keeping a pet dog and walking the dog regularly can increase the activity of elderly individuals [4]. The dog can make the owner have a strong desire to exercise because the owner must walk the dog regularly every day [4]. Taking a dog for a walk is much more frequent than the owner taking a walk alone, especially for the elderly [4]. Researchers have found that people who care for and walk dogs are healthier and end up living longer [5]. The benefits of the companionship of pet dogs to owners are worth exploring.

Billions of bacteria colonize the human intestine [6]. The gut microbiota can help the host deal with exogenous substances, such as protein, dietary fiber, and fat. The gut microbiota can produce various metabolites (such as short-chain fatty acids (SCFAs)), bacterial constituents (such as lipopolysaccharides (LPS)), and the metabolism of bile acids [6]. In addition, gut microbiota can significantly affect intestinal mucin, especially *Akkermansia muciniphila*, which can significantly improve the thickness of mucin [7]. The gut microbiota can affect host health and is associated with multiple diseases, such as diabetes and depression [8, 9]. The colonization or increase of probiotics is beneficial to the treatment of diseases [10]. However, the increase in some microbes in the intestine can promote the occurrence of diseases [11]. Therefore, maintaining the diversity and stability of the gut microbiota is an important factor in maintaining health.

Pets can affect their owner’s gut microbiota in a variety of ways. There are pet owners who enjoy interacting with their pets and even have intimate interactions, such as sharing a bed, petting, hugging, and head-to-head contact [12]. Pet owners often need to dispose of feces for their pets, which is one of the major causes of microbial changes in the environment [12]. Injury to pet owners during interaction with pets can lead to bacterial infection [12]. Recent studies have shown that keeping a cat can affect the gut microbiota of the owner [13]. Research shows that women have higher cat and dog ownership rates [14, 15]. The rate of pet ownership increases with age [15]. In addition, raising dogs promotes the movement of owners. It is worth paying attention to the effects of raising a dog on gut microbiota. In this study, we screened data on the gut microbiota of elderly individuals with a dog based on the American Gut Project (AGP). By analyzing the microbial diversity and composition of elderly dog owners, the possible effects of dog ownership on the health of the elderly were explored.

## Materials and methods

### Data sources

The data for this study came from the AGP. The AGP not only collected stool samples of participants but also collected various information, including basic information such as birth year, gender, and height. In addition, the AGP also required participants to fill in various pieces of information, including diet type, keeping pet dogs, cats, and so on. The AGP was unified following the Earth Microbiome Plan in sample collection, storage, and sequencing analysis. The AGP stored its questionnaire information and original sequencing data in the SRA database (https://www.ncbi.nlm.nih.gov/sra/) under accession number PRJEB11419. Although the AGP completed the sequencing of 25,376 samples, some of the data could not be included in this study. This study excluded people without basic information, who had antibiotic treatment within six months, recently traveled, or had severe illness. In addition, samples with a sequencing depth of less than 8000 were also excluded. Finally, we obtained data from 54 participants over 65 years of age for subsequent analysis. In addition, we randomly matched 54 samples by gender, body mass index (BMI), and age from participants who claimed not to have dogs (Table 1).

**Table 1:** Demographic and anthropometric characteristics of the elderly dog owners.

		Dog	No Dog	Chi-square	P Value
Total Number		54	54		
Age		69.78±3.15	69.72±3.18		
BMI		27.13±4.22	26.95±4.47		
	Normal Weight (Number)	20	20		
	Over Weight (Number)	34	34		
Gender	Female: Male	14:30	14:30		
Diet Type					
	Omnivore	47	44	0.007991	0.928770
	other	7	10	0.172653	0.677765

### Group

This study included 54 elderly people with dogs (ED) and 54 elderly people without dogs (END). According to body weight, they were divided into a normal-weight elderly group with dogs (NWED), a normal-weight elderly group without dogs (NWEND), an overweight elderly group with dogs (OWED), and an overweight elderly group without dogs (OWEND). According to gender, they were divided into male elderly with a dog (Male_ED), male elderly without a dog (Male_END), female elderly with a dog (Female_ED), and female elderly without a dog (Female_END).

### Converting SRA to FASTQ format

The data stored in the SRA database must be formatted. Data in SRA format need to be converted to FASTQ format. The fastq-dump.exe program (sratoolkit toolkit) was used for format conversion.

### Data processing

QIIME2 software was used to process 16S rRNA sequencing data. QIIME2 software integrated FASTQ files into a demux.qza file and then used the deblur plug-in to control the quality of the sequence data and obtain a characteristic table. Next, the "qiime phylogeny align-to-tree-mafft-fast tree" plug-in generated rootless trees. α-Diversity was obtained by using the "qiime diversity alpha rarefaction" plugin. After generating the visualized qzv file, the OTU and Shannon index were visualized in the browser. The Greengenes 13.8 databases were used to annotate and classify the microbes. OTUs that were not widely distributed (distributed in less than 1% of the samples) were deleted.

According to the characteristic table, picrust2 predicted the function of the gut microbiota.

### Statistical analysis

Microbial abundance and function prediction difference statistics were analyzed by Statistical Analysis of Metagenomic Profiles (STAMP 2.1.3). The adjusted P value was calculated using the Benjamini-Hochberg false discovery rate (FDR) method. An adjusted P value <0.05 was considered significant.

## Result

The present study was carried out to characterize dog ownership-induced changes in the properties of the owner’s gut microbiota. As shown in Table 1, age, BMI, sex, ethnicity, country of residence, and diet type were not significantly different between the dog group and without dog group.

### The effect of dog ownership on the gut microbiota of elderly individuals

**Fig 1:**
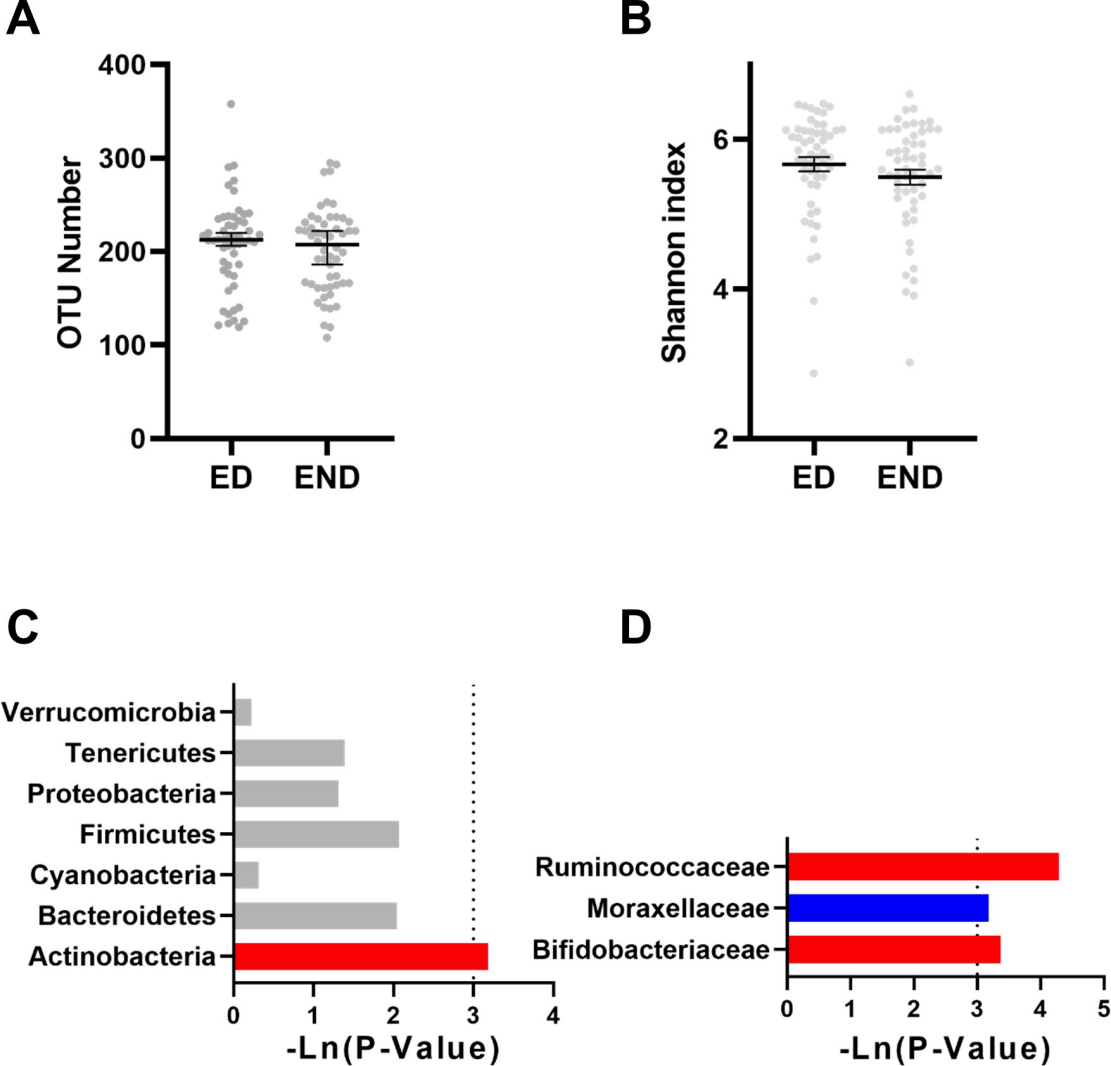
The effect of dog ownership on the microbial composition of elderly individuals. Dog ownership did not affect the (A) number of OTUs or the (B) Shannon index. The effect of dog ownership on bacteria at the (C) phylum level and (D) family level. The red bar represents a significant increase, while the blue bar represents a significant decrease.

The α-diversity analysis, which reflects the abundance and diversity of the microbial community, showed that the OTU number and the Shannon index (Shannon value was positively correlated with community diversity) were not significantly altered in the elderly with dog group compared with those of the no_dog group (Fig 1A and 1B).

As shown in Fig 1C and 1D, the microbial composition was impacted by owning a dog. At the phylum level, *Actinobacteria* were significantly increased by dog ownership (Fig 1C). At the family level, the relative abundances of *Bifidobacteriaceae* and *Ruminococcaceae* were significantly increased, while *Moracellaceae* was significantly reduced (Fig 1D).

**Fig 2:**
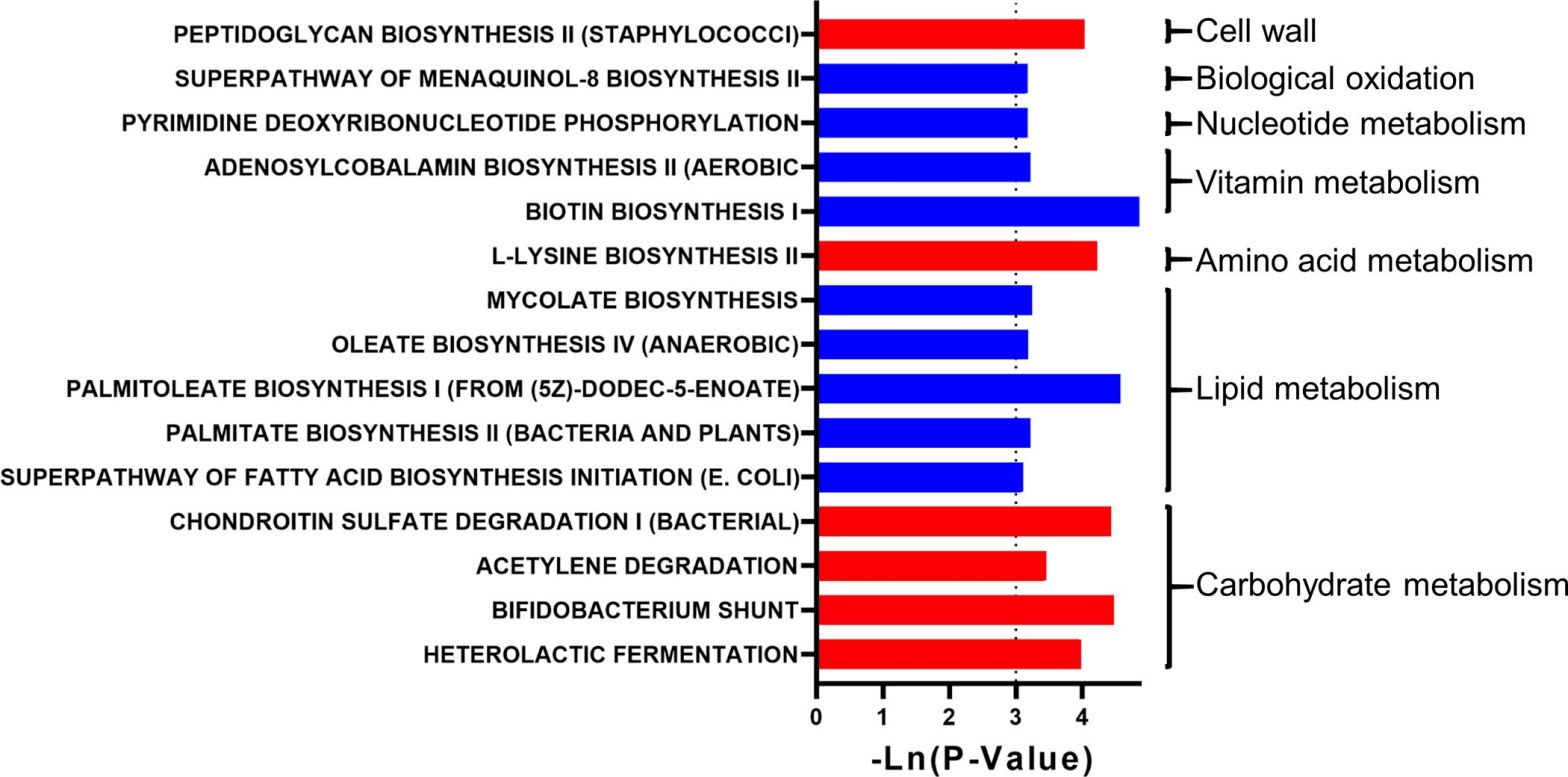
The effect of dog ownership on the microbial function of elderly individuals. The significant effect of dog ownership on microbial metabolism pathways. The red bar represents a significant increase, while the blue bar represents a significant decrease.

In addition, 11 metabolic pathways were predicted to be significantly changed (P<0.05) (Fig 2). The metabolism of carbohydrates and the cell wall were significantly increased. The metabolism of lipids, vitamins, nucleotides, and biological oxidation was reduced.

### The effect of dog ownership on the gut microbiota of normal-weight and overweight elderly individuals

**Fig 3:**
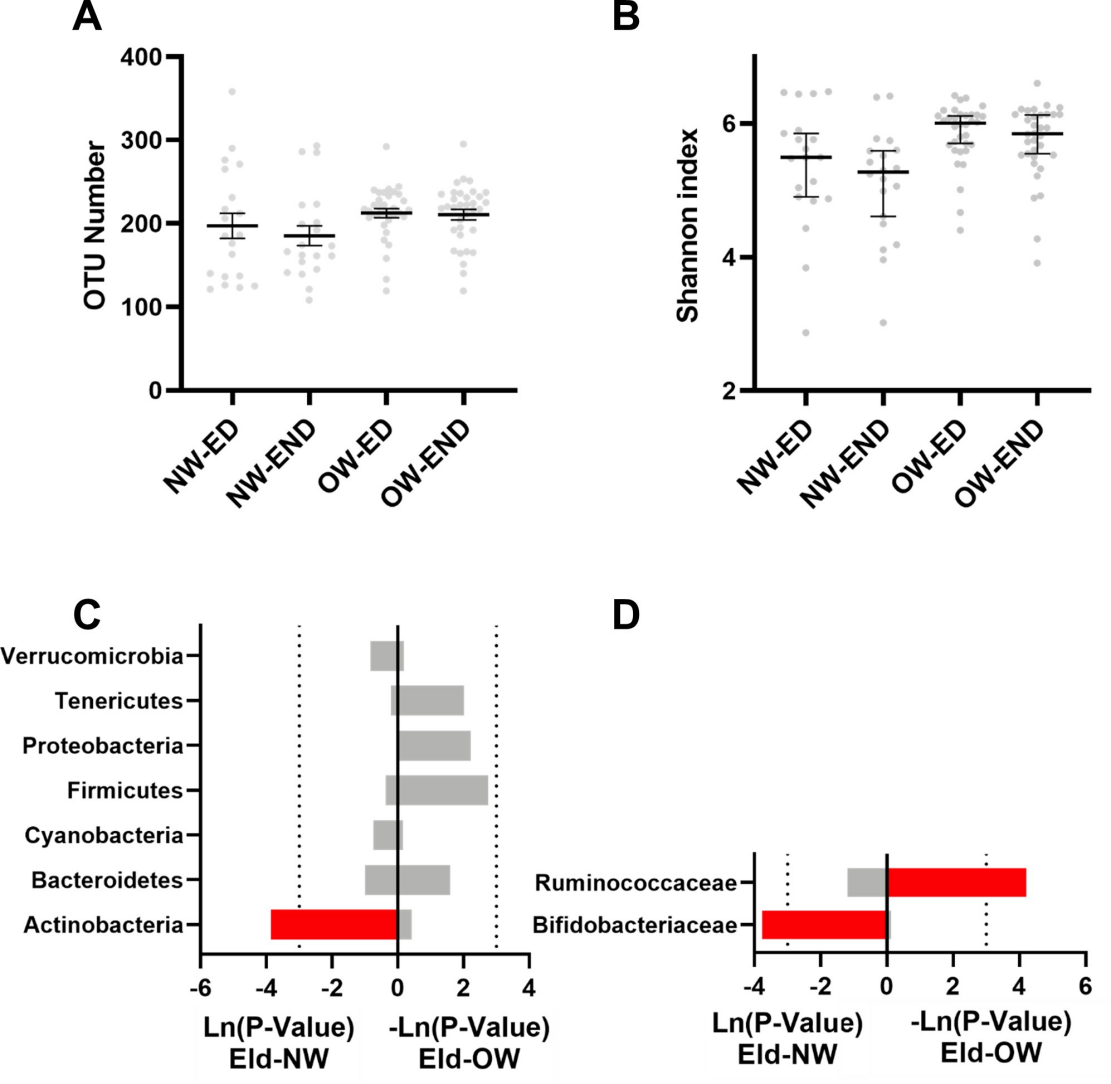
The effect of dog ownership on the microbial composition of female and male elderly individuals. Dog ownership did not affect the (A) number of OTUs or the (B) Shannon index. The effect of dog ownership on bacteria at the (C) phylum level and (D) family level. The red bar represents a significant increase, while the blue bar represents a significant decrease.

The α-diversity analysis showed that the OTU number and the Shannon index were not significantly altered in either the normal-weight elderly with a dog (NWED) or the overweight elderly with a dog (OWED) group (Fig 3A and 3B). Moreover, at the phylum level, *Actinobacteria* was significantly increased in the NWED group (Fig 3C). At the family level, the relative abundance of *Bifidobacteriaceae* was significantly increased in the NWED group (Fig 3D). *Ruminococcaceae* was significantly increased in the OWED group compared with that of the OWEND group (Fig 3D).

**Fig 4:**
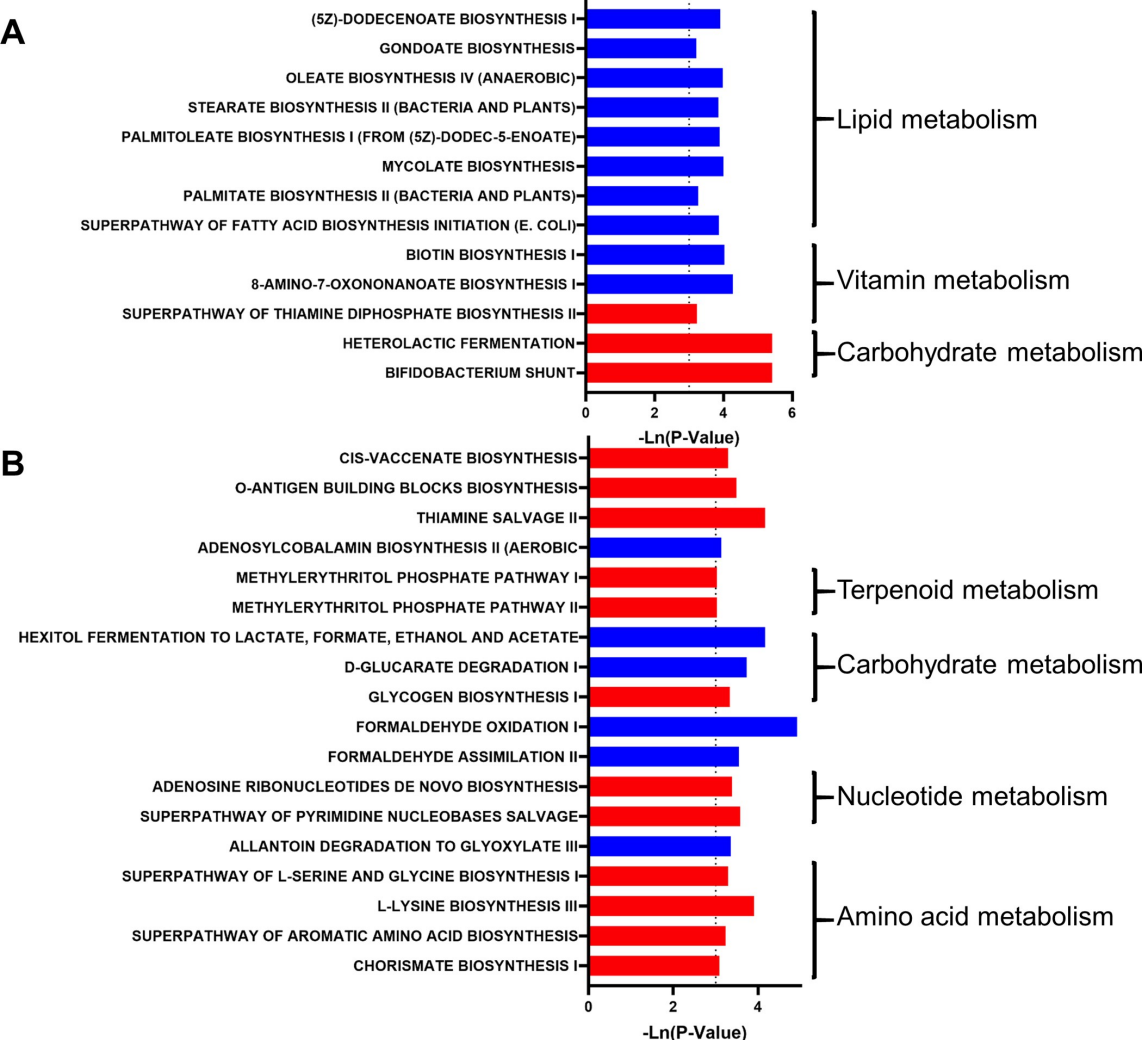
The effect of dog ownership on the microbial function of female and male individuals. The significant effect of dog ownership on microbial metabolism pathways in (A) female and (B) male individuals. The red bar represents a significant increase, while the blue bar represents a significant decrease.

In addition, 13 and 18 metabolic pathways were predicted to be significantly changed in the NWED and OWED groups, respectively (P<0.05) (Fig 4). In the NWED group, the metabolism of carbohydrates was significantly increased, while the metabolism of vitamins and lipids was significantly reduced (Fig 4A). In the OWED group, the metabolism of amino acids was significantly increased (Fig 4B).

### The effect of dog ownership on the gut microbiota of female and male elderly individuals

**Fig 5:**
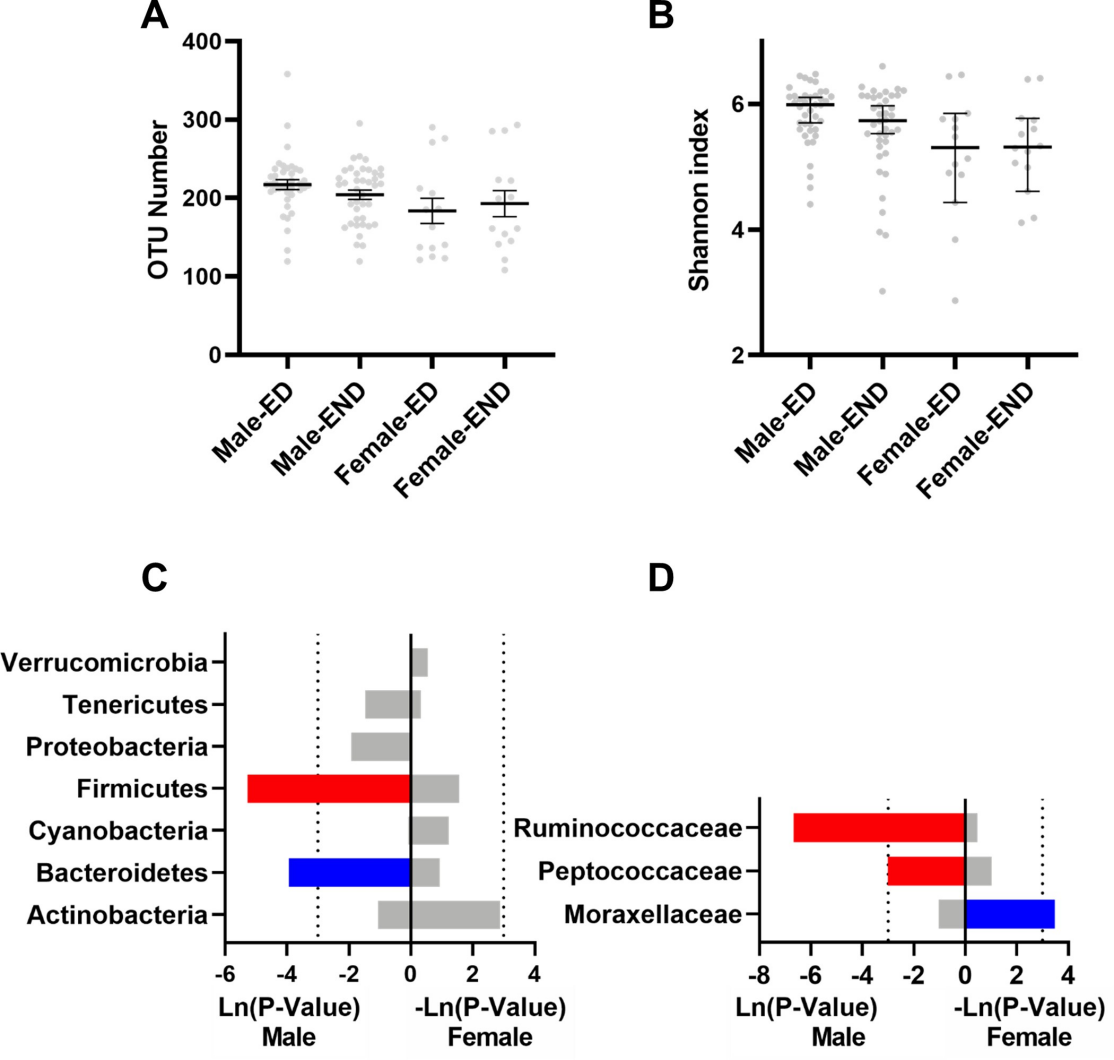
The effect of dog ownership on the microbial composition of normal-weight and overweight elderly individuals. Dog ownership did not affect the (A) number of OTUs or the (B) Shannon index. The effect of dog ownership on bacteria at the (C) phylum level and (D) family level. The red bar represents a significant increase, while the blue bar represents a significant decrease.

The α-diversity analysis showed that the OTU number and the Shannon index were not significantly altered in the female elderly groups or male elderly groups (Fig 5A and 5B). At the phylum level, the relative abundance of *Bacteroidetes* was significantly decreased, while that of *Firmicutes* was significantly increased in the Male-ED group compared with the Male-END group (Fig 5C). At the family level, the relative abundances of *Ruminococcaceae* and *Peptococcaceae* were significantly increased in the Male-ED group compared with those of the Male-END group (Fig 5D). *Moraxellaceae* was significantly decreased in Female-ED compared with Female-END (Fig 5D).

**Fig 6:**
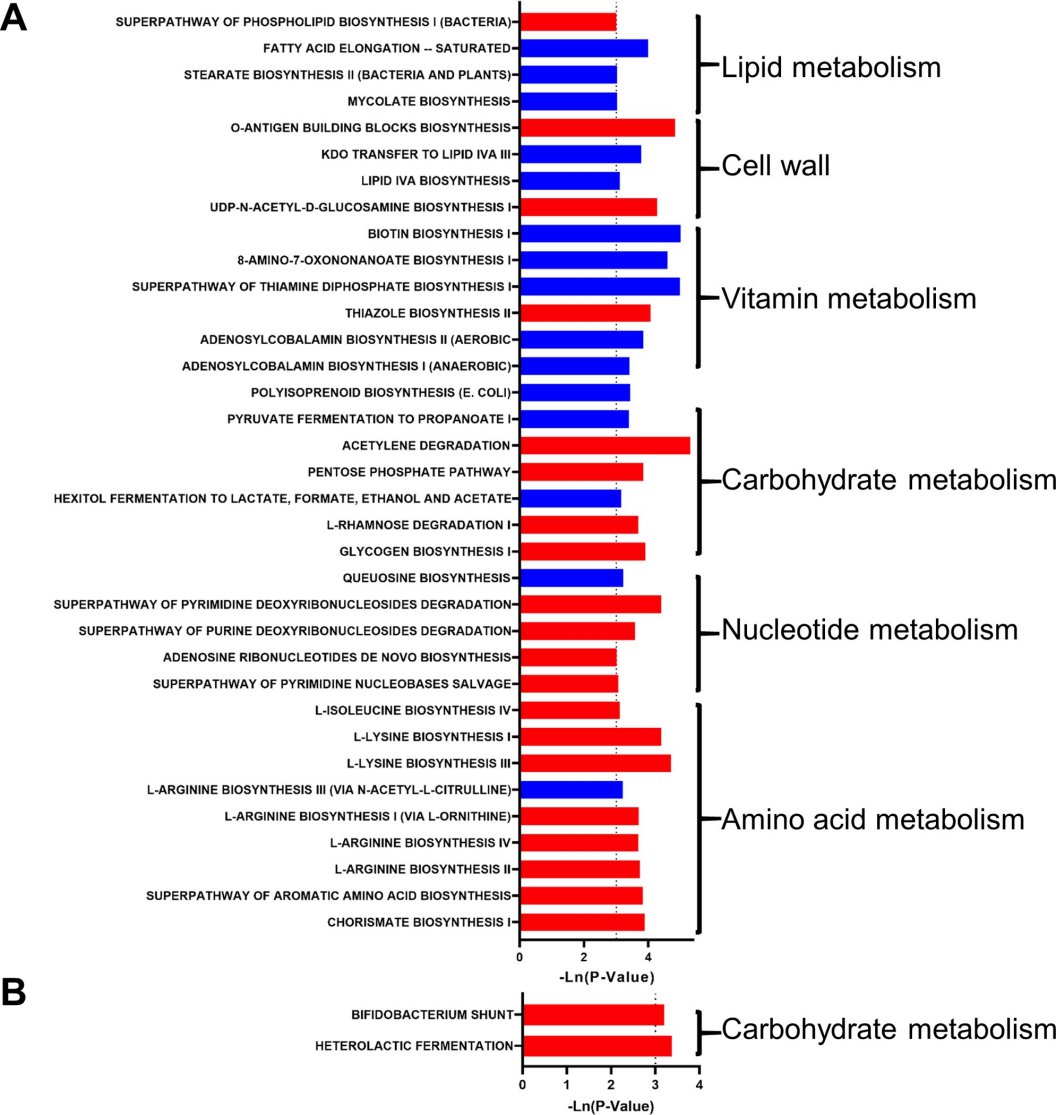
The effect of dog ownership on the microbial function of normal weight and overweight individuals. The significant effect of dog ownership on microbial metabolism pathways, (A) normal-weight and (B) overweight individuals. The red bar represents a significant increase, while the blue bar represents a significant decrease.

In addition, 35 and 2 metabolic pathways were predicted to be significantly changed in the Male-ED and Female-ED groups, respectively (P<0.05) (Fig 6). In the Male-ED group, the metabolism of carbohydrates, nucleotides, and amino acids was significantly increased, while the metabolism of vitamin synthesis was significantly decreased. In the female-ED group, the metabolism of carbohydrates was significantly increased.

## Discussion

Studies have shown that the gut microbiota is affected by the environment, such as the urban living environment and antibiotic exposure. As one of people’s closest partners, dogs affect people’s health in many ways. The present study demonstrated that dog ownership had a significant impact on the microbial structure and function of elderly individuals.

Sixty-four percent of the owners would walk their dogs for 214.1 ± 189.5 minutes per week [16]. Therefore, the exercise time of dog owners is also higher than that of others, and it is easier for them to achieve the recommended amount of physical activity [16, 17]. Regular dog walking improves physical capacity in elderly patients after myocardial infarction [18]. In addition, a study shows that the company of dogs can effectively increase the sleep time of the elderly [19]. Therefore, keeping a dog for a long time may reduce the frailty risk of the elderly [20]. Recently, a study showed that exercise plays an important role in improving the composition of the gut microbiota [21]. Thus, consistent with previous studies, dog ownership may provide benefits to dog owners in terms of gut microbiota, which is attributed to exercising with dogs.

A variety of health-promoting bacteria are present in the intestinal tract. *Actinobacteria* are the source of many important antibiotics [22]. Some *Actinobacteria* are also beneficial to humans. For example, a high abundance of *Atopodium* is beneficial to relieving depression [23]. Studies have shown that Bifidobacterium can improve metabolic endotoxemia and glucose tolerance [24, 25]. Bifidobacterium in humans has highly acquired glycosyl hydrolase encoding genes, which can enhance their metabolic ability to utilize different carbon sources consumed by the host [26]. Ruminococcaceae is the predominant bacterial family [27]. The decrease in Ruminococcaceae is associated with a variety of inflammatory reactions. Therefore, the increase in the abundance of Ruminococcaceae is considered a marker of the intervention effect [28, 29]. In addition, a high abundance of Moraxellaceae is thought to be associated with a variety of diseases, such as drug resistance, cancer, and asthma [30–32]. 16S rRNA sequencing analysis showed that *Actinobacteria*, *Bifidobacteriaceae*, and *Ruminococcaceae* were significantly increased, while *Moraxellaceae* was significantly reduced in elderly dog owners. Thus, dog ownership might promote the growth of beneficial microbes and inhibit the abundance of harmful bacteria.

Studies have reported that microbial diversity and abundance might be altered by obesity. In overweight individuals, the *Firmicutes* to *Bacteroidetes* ratio might be significantly increased, while *Bifidobacterium* abundance significantly decreased [33, 34]. In contrast, a study found that obese patients had significantly lower duodenal Firmicutes [35]. Meanwhile, Firmicutes increased with age, while Bacteroidetes decreased in elderly individuals [36]. The Firmicutes/Bacteroidetes ratio was higher in the elderly group than in the middle-aged group, but no significant difference was observed between the two groups [37]. The present study showed that the *Firmicutes* to *Bacteroidetes* ratio was increased in dog ownership groups, especially in obese individuals. *Ruminococcaceae* were significantly increased in the OW_elderly group. This result suggests that although dog ownership can increase the abundance of some probiotics, it does not significantly alter the gut microbiota of obese individuals. Obese patients need more diverse means of health control.

A growing number of studies have shown sex differences in gut microbiota in animals and humans [38, 39]. Compared with males, *Firmicutes*, the *Firmicutes*/*Bacteroidetes* ratio, and *Actinobacteria* were significantly increased in females [40]. A study showed that diet has sex characteristics for gut microbiota [41], leading to a difference in lipid metabolism [42]. In addition, the difference in gut microbiota by sex influences the occurrence and treatment effect of diseases [43, 44].

In the present study, differences in gut microbiota between male dog owners and female dog owners were analyzed (S1 Table). It was found that at the phylum level, the gut microbiota of the three phyla differed significantly, with Cyanobacteria and Firmicutes significantly increased, while *Bacteroidetes* levels were significantly decreased in elderly female dog owners compared with elderly male dog owners. Correspondingly, Cyanobacteria were also significantly increased in the gut microbiota of the no dog elderly_male group compared with that of the no dog elderly_female group. Taken together, these results indicate that *Firmicutes* and *Bacteroidetes* may be bacteria with significant differences between older male and older female dog owners. Therefore, the study revealed that dog ownership had different effects on the gut microbiota of males and females.

The dominant phyla of the dog gut microbiota are Firmicutes, Bacteroidetes, *Proteobacteria*, *Actinobacteria*, and *Fusobacteria*, which is similar to the structure of human gut microbiota [45]. Moreover, studies have shown that humans can acquire infections from their pet dogs [46, 47]. These infections are usually transmitted by scratching or biting, and mucus secretion is the main source of infection [48]. Multiple findings suggest that intimate interactions between humans and dogs are not limited to walking together, touching the head, bathing the dog, and cleaning up the dog’s excrement but also include sharing living spaces [12]. Therefore, bacteria in dog skin, saliva, urine, and especially feces may be important factors affecting the human gut microbiota. In the present study, a significant increase in the abundance of *Actinobacteria* was associated with dog ownership. Therefore, alterations in the structure of the human gut microbiota may originate from the transmission of bacteria from dogs.

This study relied more on the subjects’ self-reports, which may not be accurate. The AGP does not collect the time spent with pets, the ways they interact, the time spent walking the dog, etc. Therefore, this article cannot analyze the influence of pet dogs on the gut microbiota in more aspects. In addition, limited by the research strategy, many samples were excluded, resulting in too few samples in this study. The imbalance of the sampling makes this research not comprehensive enough. All of these issues need to be resolved by further research.

## Conclusion

This study highlights that dog ownership can promote the increase in beneficial microorganisms and suppress the number of harmful bacteria. The abundance of *Actinobacteria* was significantly affected by dog ownership. *Firmicutes*-to-*Bacteroidetes* were significantly increased in males, which is considered to be beneficial for health. Meanwhile, the probiotics *Bifidobacteriaceae* and *Ruminococcaceae* were found to be significantly increased in multiple subgroups. In addition, dog ownership may have more benefits in older normal-weight individuals and older men. However, the benefits of dog ownership on the gut microbiota of elderly individuals still need to be validated in a larger cohort.

## Supporting information

S1 Table Comparison of the gut microbiota of female with dog ownership and that of male with dog ownership. (XLSX)

S1 Graphical abstract (TIF)
