# Supplementary figures and images for: Effects of dog ownership on the gut microbiota of elderly owners
Source: PLoS One. 2022 Dec 7;17(12):e0278105. doi: 10.1371/journal.pone.0278105 (PMC9728917; doi:10.1371/journal.pone.0278105)

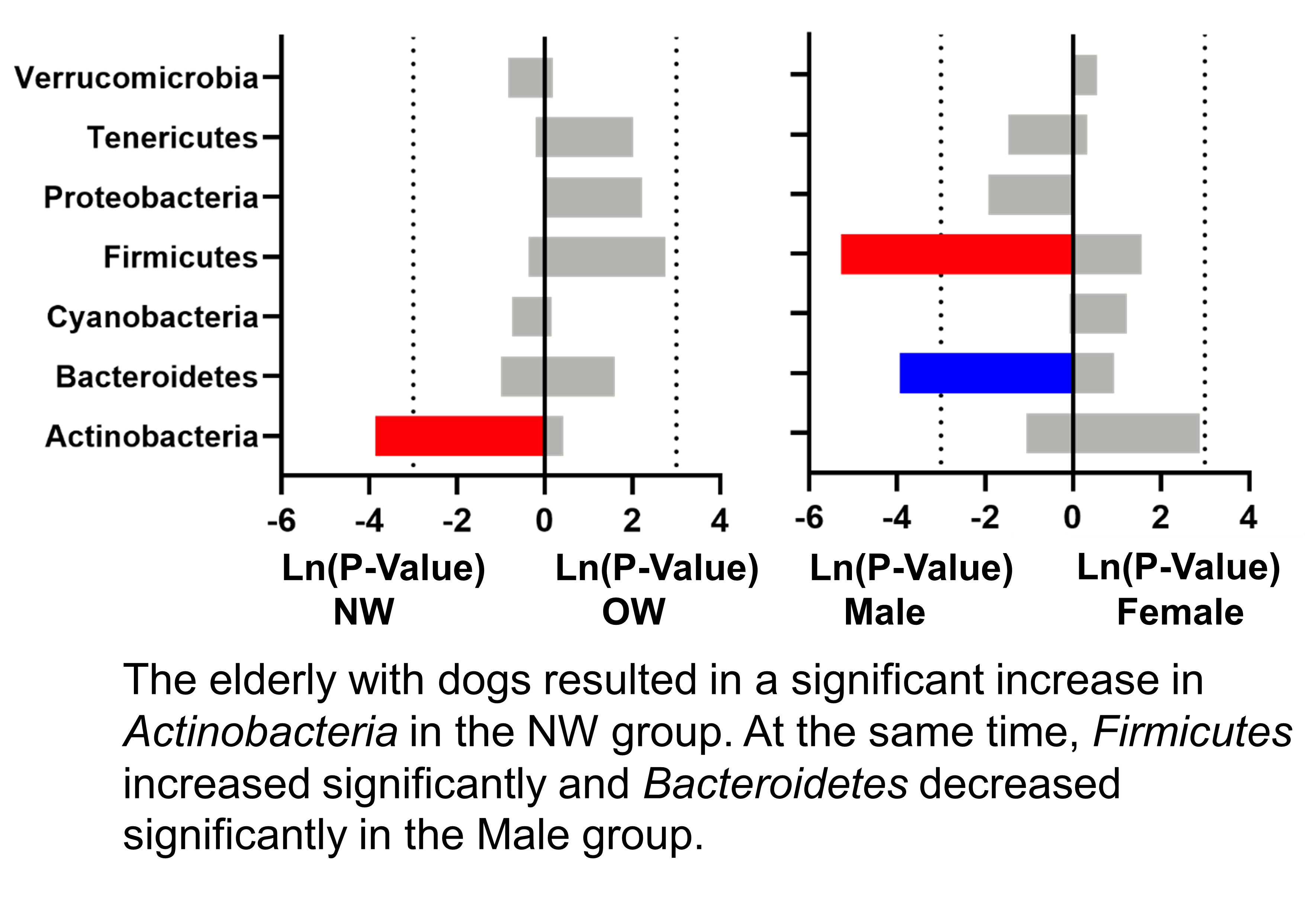

Supplement: S1 Graphical abstract — (TIF) [file pone.0278105.s002.tif]
